# Nanotheranostics: A Possible Solution for Drug-Resistant *Staphylococcus aureus* and their Biofilms?

**DOI:** 10.3390/nano11010082

**Published:** 2021-01-02

**Authors:** Dina A. Mosselhy, Mhd Assad, Tarja Sironen, Mady Elbahri

**Affiliations:** 1 Nanochemistry and Nanoengineering, Department of Chemistry and Materials Science, School of Chemical Engineering, Aalto University, 02150 Espoo, Finland; mhd.assad@aalto.fi; 2 Microbiological Unit, Fish Diseases Department, Animal Health Research Institute, Dokki, Giza 12618, Egypt; 3 Department of Virology, Faculty of Medicine, University of Helsinki, P.O. Box 21, 00014 Helsinki, Finland; tarja.sironen@helsinki.fi; 4 Department of Veterinary Biosciences, Faculty of Veterinary Medicine, University of Helsinki, P.O. Box 66, 00014 Helsinki, Finland; 5 Nanochemistry and Nanoengineering, Institute for Materials Science, Faculty of Engineering, Kiel University, 24143 Kiel, Germany; 6 Center for Nanotechnology, Zewail City of Science and Technology, Sheikh Zayed District, Giza 12588, Egypt

**Keywords:** methicillin-resistant *S. aureus*, biofilms, nanoparticles and nanofibers, theranostics, mechanisms, appraisals

## Abstract

*Staphylococcus aureus* is a notorious pathogen that colonizes implants (orthopedic and breast implants) and wounds with a vicious resistance to antibiotic therapy. Methicillin-resistant *S. aureus* (MRSA) is a catastrophe mainly restricted to hospitals and emerged to community reservoirs, acquiring resistance and forming biofilms. Treating biofilms is problematic except via implant removal or wound debridement. Nanoparticles (NPs) and nanofibers could combat superbugs and biofilms and rapidly diagnose MRSA. Nanotheranostics combine diagnostics and therapeutics into a single agent. This comprehensive review is interpretative, utilizing mainly recent literature (since 2016) besides the older remarkable studies sourced via Google Scholar and PubMed. We unravel the molecular *S. aureus* resistance and complex biofilm. The diagnostic properties and detailed antibacterial and antibiofilm NP mechanisms are elucidated in exciting stories. We highlight the challenges of bacterial infections nanotheranostics. Finally, we discuss the literature and provide “three action appraisals”. (i) The first appraisal consists of preventive actions (two wings), avoiding unnecessary hospital visits, hand hygiene, and legislations against over-the-counter antibiotics as the general preventive wing. Our second recommended preventive wing includes preventing the adverse side effects of the NPs from resistance and toxicity by establishing standard testing procedures. These standard procedures should provide breakpoints of bacteria’s susceptibility to NPs and a thorough toxicological examination of every single batch of synthesized NPs. (ii) The second appraisal includes theranostic actions, using nanotheranostics to diagnose and treat MRSA, such as what we call “multifunctional theranostic nanofibers. (iii) The third action appraisal consists of collaborative actions.

## Highlights

- *Staphylococcus aureus* is a notorious resistant pathogen that emerged from hospitals to communities, forming implant and wound biofilms.
- Nanotheranostics are “game-changers” combating this resistance and tolerant biofilms via rapid diagnosis and multimodal therapeutic mechanisms.
- Appropriate stewardship considering our three action appraisals would beat the global resistance and biofilm battle.

## 1. Introduction

### 1.1. S. aureus and Resistance Mechanisms

*Staphylococcus aureus* is a substantial etiological agent of implant-associated infections [1,2,3,4,5], skin or wound infections [6,7,8,9,10], nosocomial infections [11], sepsis, and death [12]. *S. aureus* is a dire pathogen because of its potential to acquire resistance to antibiotics [10,13,14,15]. For example, the acquisition of the *mecA* gene and production of a specific penicillin-binding protein (PBP2a) fortify *S. aureus*, resisting penicillinase-resistant β-lactams (methicillin or oxacillin) [16,17,18,19]. Apocalyptic outbreaks of methicillin-resistant *S. aureus* (MRSA) have mainly been confined within hospital settings (hospital-acquired, HA-MRSA) and to immunocompromised patients. Then, MRSA outbreaks emerged from hospitals to community reservoirs. Healthy people could contract community-acquired MRSA (CA-MRSA) [6,13,14,20,21], especially those of low socioeconomic status (lack of medical care, crowding, poverty, and intravenous drug addiction) [22,23,24], and then die [21]. CA-MRSA was reported to cause community-acquired pneumonia associated with influenza (during the 2003–2004 influenza season) in the USA, eliciting severe illness and death [25]. CA-MRSA strains are distinct from HA-MRSA in possessing a characteristic Staphylococcal cassette chromosome *mec* (SCC*mec*) type IVa and some carrying Panton-Valentine leukocidin (PVL) locus, producing PVL toxins [26,27]. PVL toxin is associated with skin and soft-tissue infections, requiring increased surgical treatment, and rarely (i.e., in the USA), it is associated with invasive pneumonia and poor prognosis [27,28]. The MRSA health problem is augmented via the simple five factors or simple five Cs, as termed via the Centers for Disease Control and Prevention (CDC) [29], facilitating MRSA transmission: (i) Crowding, (ii) skin-to-skin Contact (with colonized or infected person), (iii) Compromised integrity of skin, (iv) Contaminated fomites, and (v) Cleanliness deficiency. These factors are common, with increased risk of CA-MRSA infections among military personnel and day-care children [14,24]. The reported resistance of MRSA to vancomycin worsens the bacterial antibiotic resistance scenario [30,31,32,33,34,35]. This worse situation is because vancomycin is the bedrock of last-resort antibiotics treating notorious MRSA infections [36,37,38], besides linezolid and daptomycin [31]. If bacterial antibiotic resistance is currently a global bottleneck challenge, then the critical question here is: What are the future consequences of such bacterial antibiotic resistance? A direct answer is to return to the point of the pre-penicillin era, lack of new antibiotic classes, and the disengagement of pharmaceutical companies from the antibiotic research pipeline owing to the high economic burden of developing novel antibiotics and executing clinical trials [39,40,41]. In terms of numbers, by 2050, 10 million people would be expected to die every year globally [42,43], and more than 1.2 trillion USD would be added to world health care costs every year [44,45]. A cumulative global cost of up to 100 trillion USD would be expected if bacterial antibiotic resistance was not tackled [43]. 

What are the mechanisms behind the ruthless antibiotic resistance of bacteria? Evidence suggests that the essential mechanisms facilitating intrinsic (i.e., inherent or innate) bacterial antibiotic resistance are (i) alterations of bacterial membrane permeability and deoxyribonucleic acid (DNA), (ii) multidrug efflux pumps, and (iii) the inactivation of enzymes [46,47,48]. On the other hand, acquired bacterial antibiotic resistance is facilitated via chromosomal mutations or the acquisition of antibiotic resistance genes (horizontal gene transfer between bacteria) mediated by mobile genetic elements such as plasmids and transposons [4,49,50]. Adaptive antibiotic resistance is a transient alteration of expression of bacterial genes or their cognate proteins fostered by previous bacterial exposure to either non-lethal antibiotic concentration or successive high antibiotic doses and environmental stressors (e.g., pH, temperature, and limitation of oxygen or nutrients) [51,52]. 

### 1.2. S. aureus in Biofilms and Resistance and Tolerance Mechanisms

**Figure 1:**
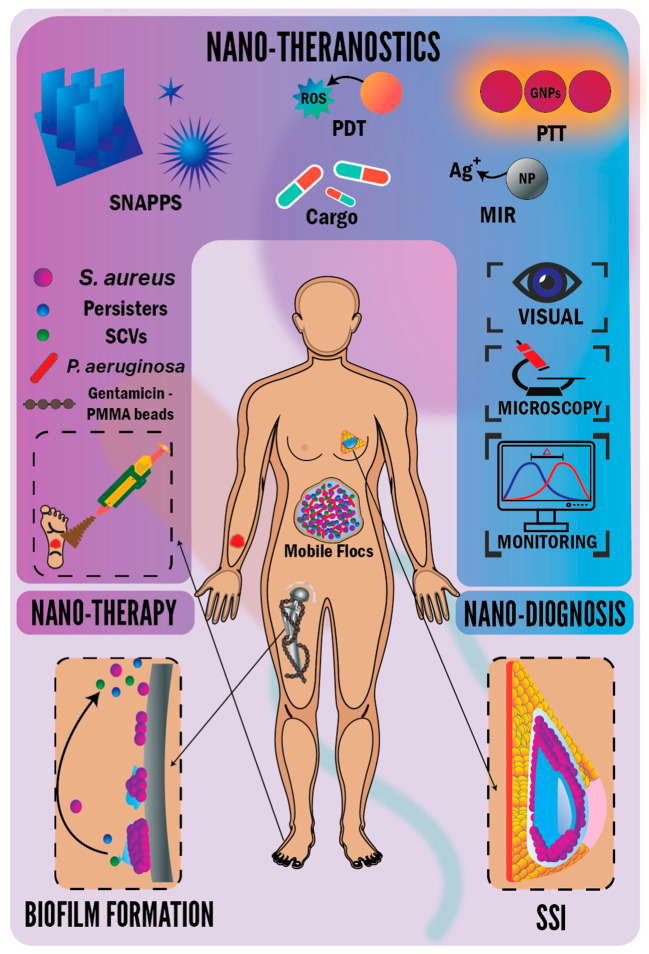
Nanotheranostics diagnose via visual detection, or fluorescence microscope, or monitoring. Nanotheranostics treat via, e.g., photothermal therapy (PTT) with gold nanoparticles (NPs) (GNPs) inducing thermal damage. Photodynamic therapy (PDT) releasing reactive oxygen species (ROS). Physicochemical properties of NPs as metal ions release (MIR), their cargo (e.g., antibiotics), and physical disruption by nanoknives or structurally nanoengineered antimicrobial peptide polymers (SNAPPs). “Multifunctional theranostic nanofibers” could be directly electrospun on a diabetic foot ulcer. *Staphylococcus aureus* associated implant (hip or breast) and wound infections. Steps of *S. aureus* biofilm formation on the hip implant are initial adhesion, microcolonies formation within an extracellular polymeric substance (EPS), biofilm maturation (3D EPS), and dispersion of planktonic cells recolonizing the implant. Surgical site infection (SSI) following breast construction could cause breast implant loss. *S. aureus* cells could adhere to each other, forming mobile biofilm flocs. Nanotheranostics perform diagnosis and therapy of *S. aureus*.

What inflames the bacterial antibiotic resistance or tolerance puzzle is the existence of biofilms in most chronic bacterial infections (i.e., medical implant- and wound-associated infections) [51,53,54,55,56]. *S. aureus* is a key biofilm producer [1,57,58,59], utilizing a system of cell-to-cell communication called quorum sensing (QS). The chromosomal locus regulating QS in *S. aureus* is called accessory gene regulator (*agr*), encoding the production of a diffusive distinct autoinducer molecule (indicating local cell density). This *agr* quorum-sensing system plays a pivotal role in the pathogenicity of *S. aureus* [6,60,61] via coordinating the architecture, bacterial growth rates, species interactions, and virulence factors (toxin production and exoenzymes) of biofilms [6,56,62]. Biofilms are sessile microbial cell communities embedded in an extracellular polymeric substance, forming a matrix and exhibiting a sophisticated altered phenotype concerning bacterial physiology, metabolism, and gene transcription [8,63,64,65,66]. The extracellular polymeric substance (EPS) matrix adheres to periprosthetic tissue and surfaces of medical implants [1,53,63,65,67] and wounds [68,69] or enables cell–cell adhesion and aggregation, forming mobile biofilms (flocs) without substrate [56,70,71], as shown in Figure 1. The matrix is composed of bacterial secreted polymers, e.g., exopolysaccharides, lipids, proteins, and extracellular deoxyribonucleic acid (e-DNA), arming bacteria with intricate three-dimensional (3D) structure and excessive resistance or tolerance against antibiotics. The e-DNA could prompt the expression of resistance genes and the horizontal gene transfer between bacterial cells within biofilms [1,51,72,73,74]. With the advent of emergent resistant bacteria and their tolerant biofilms, there is a need to explicitly elaborate on the difference between ‘resistance’ and ‘tolerance’. The term ‘resistance’ refers to a permanent genetic change in bacteria that could be acquired through point mutation or horizontal gene transfer. As a result of this permanent genetic alteration, the resistance continues with bacterial cells in biofilms and their dispersed planktonic cells. Moreover, resistance paves the way for bacteria not only to survive in the presence of drugs but also to replicate and is tied to the minimum inhibitory concentration (MIC), as a higher MIC confers a higher resistance. Comparatively, ‘tolerance’ describes a transient characteristic feature enabling bacterial cells’ survival in the presence of antibiotics (e.g., the complex lag phase of bacterial growth as a transient non-replicative evolvable phenotype tolerates antibiotics [75]) without an increase in the MIC. Tolerance is mostly linked to bacterial cells growing in the biofilm phenotype. Therefore, tolerance could be lost when biofilms are dispersed into their planktonic cells [51,56,70,71,73,76].

**Figure 2:**
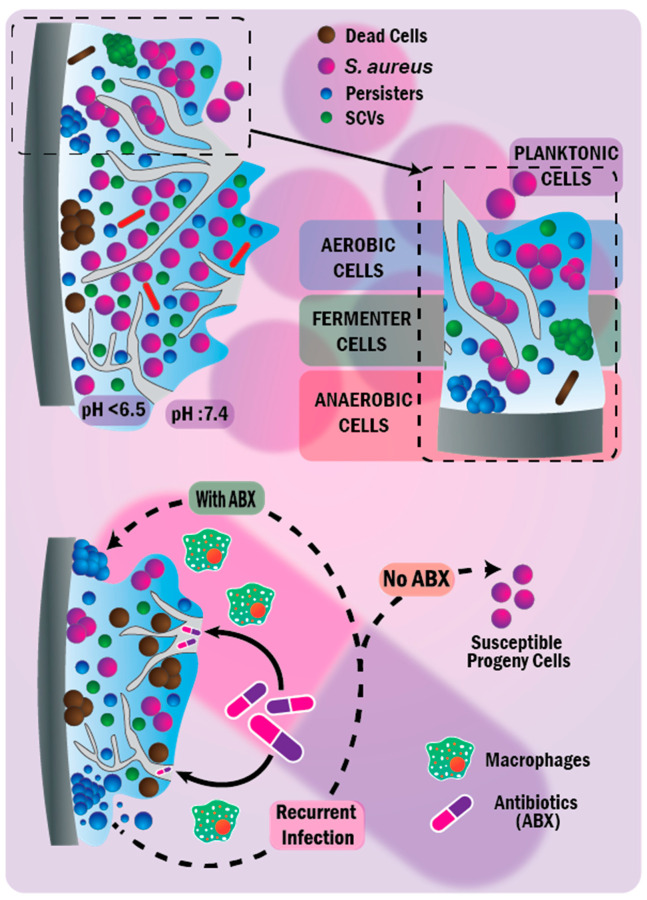
The lifestyle of *Staphylococcus aureus* biofilms (colonizing the hip implant). The biofilms are multispecies aggregates of cells, secreting a protective extracellular polymeric substance (EPS) contains open fluid channels, resembling a circulatory system. The EPS has heterogeneous compartmentalized microenvironments, resembling tumors, with different oxygen levels (aerobes, fermenters, anaerobes) and pH gradients < 6.5. Bacterial subpopulations of small-colony variants (SCVs, tolerant and resistant to antibiotics) and persisters are formed within biofilms. Persisters are antibiotic tolerant, eliciting recurrent infections, and producing progeny susceptible to antibiotics when resuming growth with no antibiotics. The biofilm EPS and embedded layers of cells constitute a physical barrier, impairing the diffusional penetration of antibiotics and invasion of immune macrophages into biofilms.

The biofilm matrix structure contains open fluid channels, resembling the circulatory system and delivering nutrients to deeply embedded bacterial cells [62,63,77]. The matrix also provides local heterogeneous, compartmentalized chemical and physical microenvironments that resemble the heterogenous compartmentalized tumor ECM [70,78]. The biofilm matrix structures are seen in Figure 2. Biofilm formation could be classified into four consecutive steps (as formed in Figure 1 on the hip implant): (i) initial bacterial adhesion to tissue or surfaces (starting as reversible adhesion; then, cells undergo species-specific behavior and secrete exopolysaccharides, changing to irreversible adhesion) via cell-surface-associated adhesins. (ii) Initial biofilm formation, where microcolonies and fluid channel architecture are formed within the produced EPS. (iii) Biofilm maturation, where EPS develops into a 3D scaffold, facilitating the formation of heterogeneous chemical and physical microenvironments. (iv) Dispersal, where a detachment of some cells from biofilms into planktonic cells, colonizing new tissue or surfaces [1,51,62,68,70,77], or cells tolerate antibiotics more than the planktonic cells [79]. Biofilms usually comprise multispecies bacterial communities (and other microorganisms), making it implausible that a single antibiotic quenches these diverse bacterial communities [55,56,72,80]. These multispecies bacterial communities are encased within an envelope (EPS matrix) and interact mostly cooperatively within the matrix and with cells of other organisms and the environment [71]. Bacterial subpopulations within the biofilm could suffer starvation or switch phenotypically after stress exposure to antibiotic treatment into slow- or non-growing persisters (persister cells). These persisters tolerate antibiotics [46,54,67,70,71,73,79,81,82] and elicit recurrent infections [53,83], as seen in Figure 2. Interestingly, persisters could also present in small proportions of non-stressed populations as an insurance mechanism to escape lethal stressors’ attack. This insurance mechanism stems from the fact that metabolically inactive non-growing cells demonstrate enhanced tolerance to antibiotics compared with their replicating counterparts [83,84,85]. Despite the stochastic development of persisters, the tendency to form them remains a genetically controlled trait [82,83,85]. This trait is controlled via HipBA (the *hip* is a high persister mutant), constituting a toxin–antitoxin (TA) locus in the workhorse model organism, Escherichia coli. Throughout normal bacterial replication, protein toxins are bound to their antitoxins (protein or RNA), inhibiting toxin activity. Throughout the persister scenario, toxins are liberated, inhibiting DNA replication and mRNA cleavage that inhibits global bacterial protein transcription and translation [85,86]. The escalating question here is: would the same TA module signaling pathway control the development of persisters in *S. aureus*? Consistent with the TA module signaling pathway in *E. coli*, Corrigan et al. [87] have also found that guanosine pentaphosphate, (p)ppGpp, was the pacemaker driving the formation of tolerant persisters in *S. aureus* via inhibiting GTPases. The inhibition of GTPases reduces the growth and amount of mature 70S ribosomes of cells and increases the antibiotic tolerance of cells. Surprisingly, Conlon et al. [88] noted that the deletion of TA modules (i.e., no increased production of (p)ppGpp) did not affect the level of the produced persisters in *S. aureus*. The unexpected finding was that persisters emanated from a stochastic process of bacterial entrance into the stationary phase and associated with a decrease in the intracellular level of adenosine triphosphate, losing energy and developing antibiotic tolerance. Persisters also have the following hallmark features. (i) The biphasic killing curve is a heterogenous non-uniform killing rate of bacteria that describes the rapid killing of most bacterial within 25 min. The remaining cells are killed after a longer time, up to 6 h [76,82,85]. (ii) The production of susceptible progeny cells to antibiotics is similar to their progenitors if they resume growth without antibiotics [76,79,89,90], as seen in Figure 2. (iii) The third hallmark feature is the heterogeneity of persisters exemplified via their bet-hedging stochastic formation. The concept ‘bet-hedging’ is an evolutionary strategy, depending on the phenotypic heterogeneity that facilitates the survival and tolerance of persisters against hefty stressful conditions [83]. In light of the biofilm broader view, biofilms possess less susceptibility to antibacterial agents and infected hosts’ immune systems than their planktonic (free-living) counterparts [1,53,55,56,62,91]. Biofilms are mostly untreatable except by surgical removal of the infected medical implant [53,64,65,67,80] or sharp debridement of wounds [55,56,68,69]. There remains little agreement on the exact resistance or tolerance mechanisms of biofilms against antibacterial agents [64,91]. There are several reasons why biofilms have such a heightened resistance or tolerance against antibacterial agents: (i) the slow and impaired diffusional penetration of antibacterial agents across the physical barrier (the matrix and layers of cells) of the biofilms [1,64,65,67,71]. This barrier is also a protective hurdle against the invasion of infected hosts’ immune macrophages into biofilms [92]; (ii) the existence of persister cells and small-colony variants (SCVs, characterized by slow growth, reduced metabolism, less motility, and associated with environmental stress, such as antibiotic treatment causing their phenotypic or genetic changes) that are tolerant and resistant to antibiotics, respectively [1,5,70,71,93,94,95]; (iii) stress-adaptive responses of bacterial cells in the biofilm and the induction of *rpoS* (RNA polymerase sigma factor)-mediated stress cause their slow growth and protection against environmental stressors and chemical agents [1,64,96]; (iv) heterogeneity of the chemical microenvironment (e.g., oxygen levels and pH gradients, ranging from <5.5 to ≈6.5 [97,98]) within the biofilm antagonize antibacterial agents [1,54,56,65,94]; (v) upregulation of resistance-associated genes in biofilms [1,56]; (vi) uptake of resistance genes through horizontal gene transfer in biofilms, i.e., plasmid conjugation and mobilization [58,71]; and (vii) the multispecies bacterial communities within the biofilm consortia [56,70]. 

### 1.3. Nanotechnology Offers Diagnostic and Antibacterial and Antibiofilm Properties

There is a robust demand to research novel antibacterial agents that defeat the global antibiotic resistance crisis to avert returning to the pre-penicillin era [46] or entering the post-antibiotic era, where common bacterial infections could kill [99,100]. This demand includes novel multi-targeted antibacterial agents effective against multi-(bacterial species and faceted properties) of biofilms [53,59,70,80]. Nanoparticles (NPs) could combat superbugs due to their unique physicochemical properties, generating heightened therapeutic effects against planktonic cells and biofilms [74,101]. The fundamental three antibacterial mechanisms of NPs are central to (i) induction of oxidative stress through the production of reactive oxygen species (ROS) that disrupt bacterial membranes, inactivate essential proteins and enzymes, and express oxidative proteins. (ii) Release of metal ions, interacting with functional groups (e.g., phosphates, sulfhydryl, carboxyl, amino) of DNA and proteins and disrupting their physiological functions. (iii) Induction of non-oxidative stress through the interaction of NPs with bacterial cell membranes, resulting in cytoplasmic leakage [47,74]. The main antibacterial prowess of NPs is that these antibacterial mechanisms could coincide, decreasing the probability of bacteria to develop resistance because multiple simultaneous bacterial gene mutations would be required [47,101,102]. Therefore, antibacterial NPs could achieve the outstanding concept of ‘resistance-resistant’, where multi-target bacterial inhibition occurs, and NPs become more resistant to resistance development. This concept would be a technological leapfrog rather than discovering novel antibiotics, which could be a daunting process and an economic burden [103]. Considering the pioneering properties of antibacterial NPs, how could these properties have a pivotal role in inhibiting or eradicating the biofilm dilemma? To date, the fundamental answer is the size of NPs (5 to 200 nm) that allow their penetration through the fluid channels (10 nm to few micrometers) of biofilms [74]. Even though more than a hundred years have elapsed since Paul Ehrlich was awarded the Nobel Prize, his concept of developing “*magic bullets*” still dominates a significant area of interest within the field of pharmaceuticals [104,105]. This domination stems from the promising therapeutic efficacies and the minimal drawbacks of these magic bullets because they specifically target certain bacteria, killing them with a few harmful side effects on other body tissues [105]. By contrast, biofilm infections are similar to a fortress of multispecies bacterial communities and multifaceted physical and biological properties, providing shelter against single “magic bullet” strategies [70]. In terms of targeted drug delivery, NPs could also be used as potential drug carriers, facilitating targeted delivery and the controlled release of drugs [47,102]. 

If NPs could combat the bacterial resistance and biofilm dilemma, then how would nanotheranostics potentially revolutionize medicine? It is necessary here to first clarify what is meant by theranostics. The term “theranostics” is commonly referred to as a combination of diagnostic and therapeutic properties into a single component [106,107] and emerging as a powerful platform toward personalized medicine [106,108]. Speaking on the aimed personalized medicine, it extends beyond the warm, caring manner to patients [109] and the traditional idea of one drug fitting all patients toward administering the specific drug to the right patient at the right time [108]. The availability of a single agent for diagnosis and treatment is at the heart of challenges facing the transfer of theranostics into the clinical field; however, nanotechnology can cross this challenge by entangling diagnostics and therapeutic agents [108]. Diving more specifically into the theranostic nanomedicine, it encompasses the NPs tagged by labels or possessing intrinsic physicochemical properties that can diagnose the disease and induce treatment themselves or through their carried cargo (e.g., chemo-, radio- or gene therapeutics or combinations of them) [110,111]. Tagging bacterial-derived antigens with NPs ameliorates the signals detected from the targeted specific binding of even low bacterial concentration and forms a thorough diagnostic probe [112]. The early diagnosis and treatment of diseases are priceless because at the earliest stage, the diseases are curable or at the least treatable [107]. Therefore, theranostics facilitate superior control of the diseases [111], increase therapeutic efficacy, and avoid the drawbacks [108,111]. 

In contrast to extensive theranostic research on different cancer types, there is much less information about theranostic effects against bacterial infections and their biofilms, which also pose a growing public health concern worldwide. However, antibacterial nanotheranostics can play a pivotal role in tackling this bacterial antibiotic resistance quandary and their biofilms via detecting (sensing) the presence of bacterial infection and consequently facilitating treatment [111]. Nanotheranostics could provide prowess rapid and accurate bacterial identification, helping to cross the hurdle of a low number of pathogens at early infectious stages [112] and the long (i.e., two days) traditional antibacterial susceptibility tests. Consequently, nanotheranostics could achieve higher success treatment rates [108,112]. Previous review articles [106,107,108,110] focusing on understanding the powerful impact of developing nanotheranostics for chemotherapy and different biological applications do exist. However, the exact nanoparticles’ antibacterial working mechanisms are still not known. This comprehensive review generates fresh insights into the detailed antibacterial and antibiofilm therapeutic mechanisms of nanotheranostics. We elucidate such mechanisms after providing a deeper understanding of *S. aureus* resistance and biofilm (lifestyle, mysteries surrounding it, and its resistance and tolerance) in a grasping narrative context supported by our constructed informative, artistic figures. Therefore, this review makes several significant contributions to the more in-depth understanding of the emergent resistant bacterial infections, specifically MRSA and their tolerant biofilms associated with implants (orthopedic and breast) and wounds. The present review explores the critical roles of different antibacterial nanotheranostics in curbing bacterial resistance and biofilm dilemmas. We provide essential insights on the challenges dampening the progress of such antibacterial nanotheranostics that are usually overlooked in studies. Ultimately, we reflect on our critical perspectives, highlight research gaps, and answer the big question in this field: How can we solve *S. aureus* resistance and its tolerant biofilms? 

## 2. Implant-Associated Infections 

One of the major problems ensuing orthopedic implant surgery is a bacterial infection, resulting in osteomyelitis and prosthetic joint-associated infections (PJIs) [113]. Gentamicin is an aminoglycoside antibiotic [114] with potent bactericidal activity against Gram-negative bacteria and the Gram-positive *S. aureus*, which is the most vicious bacterial infection associated with osteomyelitis and PJIs [115]. Although gentamicin is thermally stable [113,116], its main disadvantages are its nephrotoxicity, ototoxicity [114], and poor penetration into deep bone surgery sites. Therefore, local treatment would attain the stunning advantages of gentamicin and avoid its disadvantages [113,116]. Multimodal treatment could outpace orthopedic implant-associated infections. This multimodal treatment comprises systemic antibiotic administration, local slow-releasing gentamicin-loaded beads, and the debridement of necrotic and infected tissue [116,117,118]. This local gentamicin therapy crucially supports systemic antibiotic administration, preventing orthopedic implant-associated infections [119], and it remains important to achieve concentrations that exceed the MIC of infecting pathogens [118]. Regarding debridement, the resulting bone defects could be either filled with autologous (bone or muscle) tissue or with implants (beads), such as polymethylmethacrylate (PMMA) [113]. PMMA beads loaded with gentamicin have been clinically applied in practice over the past 45 years, incorporating gentamicin between chains during the exothermic polymerization process [113,116,118]. Gentamicin release from PMMA beads is a diffusion phenomenon classified into two phases. First, an initial burst release lasts from minutes to hours following implantation, where the surface gentamicin dissolves from PMMA into body fluids. Second, a prolonged, slower sustained release lasts from several days to years, where water-soluble gentamicin diffuses from the PMMA (hydrophilic material attracting water molecules) following the in-depth penetration of water containing body fluids [118,120]. The main issue pertinent to PMMA beads is the necessity for secondary surgery to remove the non-absorbable beads. The beads could also act as a surface for the colonization of secondary bacterial biofilms after releasing gentamicin [116] and specifically if the released concentration is insufficient (sub-lethal concentration) [118]. Sub-lethal antibiotic concentrations drive the formation of *S. aureus* biofilms and offer a chance for biofilm cells to switch into persisters [79]. Several other attempts have been made to avoid the drawbacks of gentamicin and PMMA beads, such as the attempt of Moghaddam et al. [121] using Expert Tibia Nail (ETN) PROtect™ coated by a biodegradable gentamicin-laden polymer, which does not release gentamicin into the circulation, avoiding its systemic drawbacks and preventing osteomyelitis [121]. However, if biofilms are formed, the shortage of oxygen and nutrients within them makes bacteria tend to shuffle into anaerobic metabolism, increasing the acid production that reduces the activity of gentamicin [113,114]. Moreover, the slow, sustained gentamicin release from the beads incurs the development of SCVs. *S. aureus* SCVs were isolated from four patients with a history of previous treatment with gentamicin beads. The treatment strategies for patients with SCVs end in failure, even though the administered antibiotics were effective in vitro against *S. aureus* progenitors (i.e., MICs of gentamicin ramped up to 32-fold higher for SCVs) and recurrent infections. Conversely, patients with no SCVs suffered no recurrent infections [117]. Tackling the puzzle of SCVs, two *S. aureus* (wild-type and SCV) strains were recovered from a patient with an implanted knee suffering PJI during the acute and recurrent phases. The resistance of *S. aureus* SCVs overweighs the resistance of the wild-type strain via multi-complex protective approaches against the host inflammatory and oxidative responses. Examples of such protective approaches of SCVs are (i) possessing a stringent response characterized by an inhibition of DNA replication and post-transcriptional regulation via decreased ribosomal assembly, inhibiting bacterial cell growth. (ii) Virulent expression of toxins, including phenol soluble modulins (PSM, causing both the evasion of *S. aureus* to host neutrophils and indolence within host osteoblasts), alpha- and delta-hemolysins, and the *agr* locus. (iii) Upregulation of genes detoxifying the host oxidative stress response. (iv) Downregulation of genes involved in the cell wall regulon *vraRS,* orchestrating antibiotic resistance and switching SCVs from acute to chronic infections [5]. A more dramatic PJI was reported in a patient with an implanted right hip prosthesis, where his left hip was previously replaced with 60 gentamicin-impregnated cement beads. His right hip prothesis was loosened and, consequently, replaced. The dramatic scene was the isolation of gentamicin-resistant Staphylococcal strain from the hip prosthesis. Then, the authors questioned the usefulness of using gentamicin-impregnated cement beads. Did the used beads help the patient or stimulate the development of the gentamicin-resistant *Staphylococcus* strain [122]? The suspicions about the efficacy of administrating gentamicin-releasing cement were summarized as such cement adversely contributes to the development of gentamicin resistance because of the insufficient sub-lethal released concentrations [122,123]. Another problem that has recently been addressed regarding local gentamicin therapy following knee implant was the ‘hidden’ gentamicin allergy, causing pain and swelling of the knee, and allergic contact dermatitis [124].

The human breast contains endogenous flora originated from the nipple ducts, similar to that of the healthy skin [3]. Staphylococcal skin flora are commensally resided and commonly cultured from surgical sites following breast reconstruction (BR) [20]. The patient’s skin is the predominant source of infection at the time of surgery. Surgical site infections (SSIs) could worsen this infection scenario, provoking breast implants’ loss (as displayed in Figure 1) and costing healthcare systems [125]. Multidrug-resistant bacteria, causing nosocomial infections, are the most common implant-associated infections [1]. Postoperative infections have existed as a challenging complication following BR. Surgical procedure (i.e., surgical environment, contaminated saline, contaminated implant during surgery) and the underlying clinical condition of the patient are the main contributing factors for breast implant-associated infections [3]. Regarding orthopedic implants, bacterial antibiotic resistance made routine surgical procedures, such as hip and knee replacements, a looming challenge because of a foreseeable risk of contracting a life-threatening untreatable bacterial infection [4,103]. However, the infection rates in orthopedic implants (an incidence rate of 1.5% [126]) remain lower than in breast implants, which have an incidence rate ranging widely between 1% and up to 35% [127], resulting in breast explantation and making it compelling to adopt a series of precautions at BR to reduce such a high infection rate. The assessment of 121 breast implant procedures from seven breast units operated by 22 surgeons revealed inconsistent prophylactic precautions. What stood consistent was the MRSA screening and prophylactic perioperative administration of antibiotics. The authors concluded that a breast implant checklist for infection prevention could be a better practice [128]. Barr et al. [125] have set a “Theatre Implant Checklist” to prevent SSIs in BR, including four main points. (i) Pre-operation, has MRSA screening been performed and treated if positive? (ii) Upon surgery induction, there is a series of checkpoints as has the patient received prophylactic antibiotics? Has a conductive warming blanket been placed? Have no entry signs been put on doors, and laminar flows been operated? (iii) Upon implantation, the following should be checked. Have the implant pockets been washed, and the surgeons changed their outer gloves before implant handling? (iv) Post-operation, have postoperative antibiotics been prescribed? Although Hart et al. [20] found a low incidence of MRSA colonization (<5%) postoperatively following BR, they pointed out an association between postoperative MRSA colonization and the delayed wound healing. This drawn association arose because MRSA carriers showed a higher incidence of major complications as postoperative SSI followed by delayed wound healing. This delayed wound healing was explained by MRSA colonization’s natural sequelae to contaminate wounds and subsequently impair healing. More recently, Agochukwu et al. [129] reported the most extended case of a late bilateral breast MRSA infection after 19 years of breast augmentation in a 42-year-old woman. The case had a history of intravenous drug abuse, causing the hematogenous spread of the transient bacteremia to end with concurrent breast and sternal infections. The adopted treatment regime was explantation and long-term intravenous vancomycin.

## 3. Wound-Associated Infections

The skin stays the largest and most exposed human body organ [6,8,130,131], shielding the body against the penetration of pathogenic bacteria [131,132,133]. There are two primary components of the skin: the outermost epidermis (epithelial component) and the underlying dermis (connective component) [6,131,133,134]. Skin epidermis includes the interfollicular epidermis, hair follicles, and several stratified layers of differentiated keratinocytes, melanocytes, and Langerhans cells (dendritic immune cells) [131,134,135]. These layers of differentiated keratinocytes are mainly responsible for the physical barrier function of the epidermis [133,134]. Skin dermis includes innate cells (i.e., macrophages, dendritic cells, and mast cells), innate lymphoid cells, and many lymphocytes, which mediate the immunological function of skin, including phagocytosis or killing of invading bacteria that breach the skin [134]. Moreover, preventing pathogenic bacteria from penetrating through and dwelling epithelial surfaces could be a collaboration of two other approaches. First, the non-specific immunity of skin (acidic pH, lipid density, and scarce nutrients) orchestrates a harsh environment against pathogen invasion. Second, the competition between the commensal microbes colonizing cutaneous surfaces and invading bacterial pathogens is achieved via secreting novel natural products [6,130], highlighting this concept as “colonization resistance” [8,62,134]. On the other hand, all open wounds lack a protective skin barrier and contain endogenous (skin flora of patient) or exogenous bacteria, which are initially killed by the patient’s immune system. However, suppose the bacteria are attached to the wound surface and proliferated to a mature biofilm, resulting in a biofilm-infected wound [62]. In that case, that remains the most common barrier hindering wound healing [8,68]. 

Even though inflammatory cells (such as macrophages) at the wound site incur tissue repair and regeneration, dysregulation of the inflammatory response can plausibly occur, converting the wound into chronic [136]. Chronic wounds referred to non-healing wounds from 30 days or more [68,137]. Notable examples of chronic wounds are diabetic foot ulcers, pressure injuries, venous stasis ulcers, and ischemic ulcers [68,137,138,139]. Chronic wounds also fuel the formation of biofilms because necrotic tissue and debris are niches for bacterial attachment. Compared to acute wounds, chronic wounds are more vulnerable to infection because of compromised immunity of patients [55,68], reduced susceptibility to antibiotics and greater activation of inflammatory responses [56]. Biofilms in chronic wounds are also a culprit for “hijacking” the patient’s immune response [138]. This hijacking is facilitated via expressing superantigens (e.g., enterotoxins and toxic shock syndrome toxin-1 of *S. aureus*), enabling a final release of pro-inflammatory cytokines from T lymphocytes [138,140] and so manipulating the immune response of the patient to be in a perpetual state of hyper-inflammation. In this hyper-inflammation state, the patient cannot conquer biofilms [138]. A previous study [137] has established a clear-cut more prevalence of biofilms in chronic wounds (60%) than in acute wounds (only 6%). To further perplex biofilms in chronic wounds, multispecies bacterial communities are predominant in biofilms in chronic wounds [68], and limited oxygen in deeper embedded cells of biofilms orchestrates the proliferation of anaerobes [56]. Previous studies have confirmed that *S. aureus* is the most prevalent universal bacterial insult to chronic wounds [37,141,142,143]. Sievert et al. [144] have identified a case of seven patients infected with vancomycin-resistant *S. aureus* (VRSA) in the USA. All infected patients embraced chronic underlying conditions, such as chronic skin ulcers and diabetes, a history of infection with MRSA and vancomycin-resistant enterococci (VRE), and a previous treatment with vancomycin. The authors suggested that VRE transferred the *vanA* gene to *S. aureus* via plasmid conjugation. This *vanA* gene transfer was proven [15] in studies that isolated VRSA from a polymicrobial biofilm colonizing indwelling nephrostomy tube, where VRE (*Enterococcus faecium*) donated the non-integrated *vanA* gene to MRSA. Neopane et al. [9] demonstrated that *S. aureus* isolated from wounds of hospitalized patients possess a spectacular ability to form biofilms and multidrug resistance (86.7% of isolates), where 43.3% of *S. aureus* in biofilms were identified as MRSA. It has also conclusively been shown that [145] *S. aureus* in biofilms halt wound healing via delaying wound re-epithelialization. 

## 4. Antibacterial Nanotheranostics

Investigators have recently examined the properties of nanotheranostics, including antibacterial agents such as antibiotics, NPs, antibacterial peptides or polymers (i.e., star-shaped polymers), photodynamic therapy (PDT), and photothermal therapy (PTT) [111], as shown in Figure 1. PDT is generally understood to mean a kind of therapy involving the targeted delivery of a photosensitizer (PS) to the infected site and being irradiated with light of a specific wavelength, consequently activating the PS to produce ROS damage the infected cells. PTT refers to administering a probe, absorbing light, and transforming it into heat that destructs the infected tissue [111].

### 4.1. Theranostic Nanoparticles (NPs) and Nanofibers 

Nobel metal NPs, such as silver (Ag), gold, and platinum, have long been used as therapeutic antibacterial agents [102,112]. Gold NPs (GNPs) could specifically be tied with therapeutic drug delivery and diagnostic applications because of their unique properties (e.g., facile preparation, surface functionalities, tunable core size, photothermal, and surface plasmon-related optoelectronic properties, inertness, and biocompatibility) [112]. Semiconducting NPs (defined as quantum dots, QDs), such as zinc sulfide, cadmium sulfide, and zinc oxide, have become vital tools in the diagnostic bioimaging and therapeutic drug delivery because of their size- and shape-dependent optoelectronic properties and high surface area to volume ratios [112,146]. Mesoporous silica NPs (MSNs) provide massive thrust to the area of antibacterial drug delivery, loading up to 10-fold more drug than non-porous silica NPs [74]. The available tuning of the core size, surface functionalities, and structural features of MSNs (e.g., high surface areas and tunable nanometer-scale pore sizes) make them a groundbreaking system that optimizes multifunctional therapeutic controlled drug release and diagnostic imaging modalities [102,147]. MSNs could palpably deliver the antibiotic cargo in a targeted manner, achieving the required antibiotic concentration at the infected tissue and reducing the drawbacks of systemic antibiotics [102,148]. 

#### 4.1.1. Nanoparticle-Mediated Diagnosis of *S. aureus*

**Figure 3:**
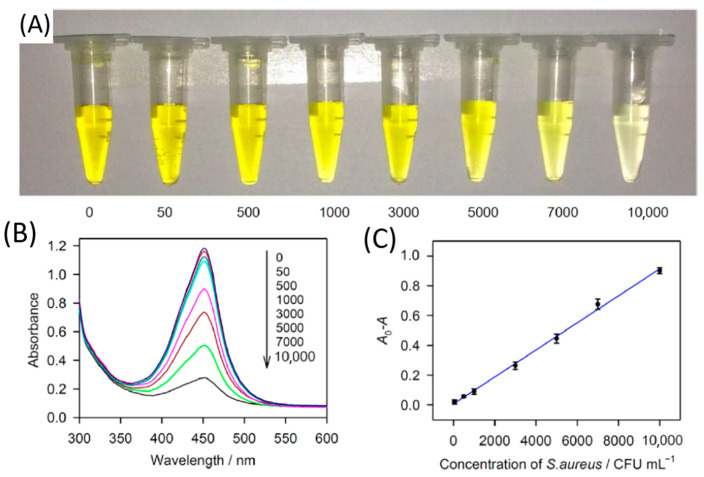
Nanodiagnostics of *Staphylococcus aureus*. Selective colorimetric method for detecting *S. aureus*, fading the yellow color with the increased *S. aureus* (colony-forming unit (CFU)/mL) (**A**). UV-vis absorption spectra, showing the diminishing of the absorption peak (at 450 nm) of copper-based metal–organic framework nanoparticles with the increased concentration of *S. aureus* (**B**). Changes of the absorbance at 450 nm of the resulting solutions for detecting the different concentrations of *S. aureus* (**C**). Reprinted with permission from [151]. Copyright, 2017, American Chemical Society.

Aptamers are small artificial single-stranded nucleotides (10 to 100) that bode well in the efficient binding and detection of bacterial targets. In NP-mediated colorimetric aptasensors, NPs could assist the capturing aptamer (the bioreceptor detects and binds the target) and participate in signal conversion (by transducer or probe) [149]. Chang et al. [150] developed an easy and low-cost method to accurately detect *S. aureus* using aptamer-conjugated GNPs (60 nm) followed by a bead-based amplification. They used a light scattering-sensing system to detect the amplified GNPs, detecting single bacterial cells within 1.5 h. Wang et al. [151] also developed an easy, sensitive, and selective colorimetric method for the detection of *S. aureus*. They combined copper-based metal–organic framework (Cu-MOF) NPs (size of 550 nm) modified with *S. aureus* aptamer and Fe_3_O_4_ modified with *S. aureus* aptamer. The method is based on the peroxidase-like activity of Cu-MOF NPs catalyzing 3,3′,5,5′-tetramethylbenzidine in the presence of H_2_O_2_, producing a yellow color. In the presence of *S. aureus*, the aptamer-modified Cu-MOF and Fe_3_O_4_ NPs specifically bind the surface of *S. aureus* cells. Following magnetic separation, Cu-MOF NPs bound to bacterial cells would be removed from the supernatant, decreasing the number of Cu-MOF NPs in the supernatant and so fading the yellow color with the increased concentration of *S. aureus* (as depicted in Figure 3). 

#### 4.1.2. Nanoparticle-Mediated Therapeutic Antibacterial and Antibiofilm Actions 

**Figure 4:**
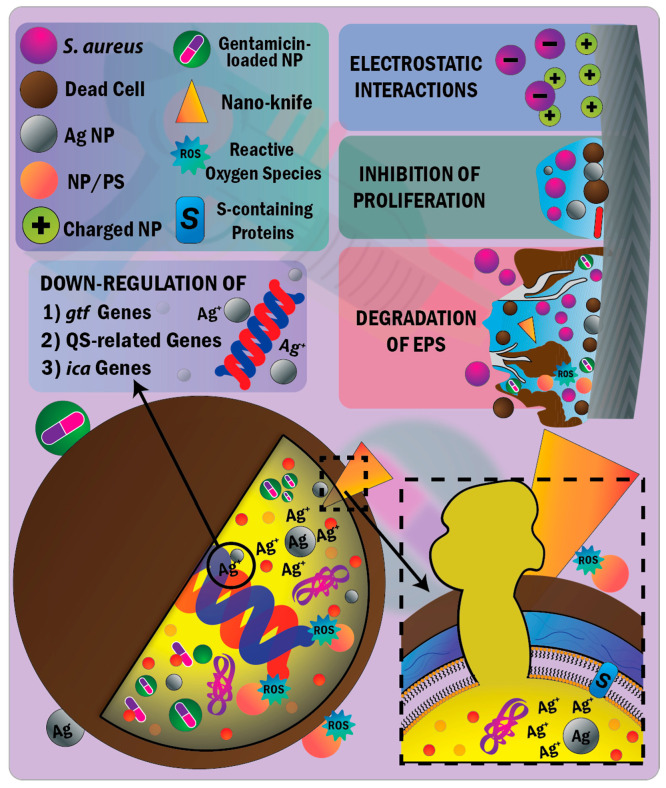
Therapeutic antibacterial and antibiofilm mechanisms of different sizes and shapes of nanoparticle (NP) gunshots. Antibacterial mechanisms are started by large contact areas between the bacterial surface and small-sized NP shots, hitting bacteria forming pits, inducing ROS (damaging membranes), and releasing metal ions (e.g., silver ions, Ag^+^). The positively charged ions (Ag^+^) interact with negatively charged groups (e.g., sulfur, thiol, and phosphorous) in proteins and enzymes, and deoxyribonucleic acid (DNA), inactivating proteins and enzymes and inhibiting replication, respectively. Ag NPs or Ag^+^ inhibit the electron transport chain, collapsing respiration. Gentamicin in drug-delivery systems inhibits protein synthesis, binding to the ribosomal 30S subunit. The antibiofilm mechanisms are started by electrostatic interactions between NP shots and bacterial surfaces, preventing surface adhesion. NP shots or Ag^+^ downregulate the expression of glucosyltransferase (*gtf*) genes, quorum sensing (QS)-related genes, and *ica* (intercellular adhesion) genes, inhibiting biofilm formation and proliferation. NP shots or activated photosensitizer (PS) induce ROS, degrading the extracellular polymeric substance (EPS) of mature biofilms. The antibacterial mechanisms of NPs also kill bacterial cells in biofilms.

This review pioneers our understanding of the mechanisms behind the antibacterial therapeutic effects of NPs. We will begin with the mechanistic story and address a series of confusing questions to elaborate on the different mechanisms as delineated in Figure 4.

Assuming that Ag NPs or NPs, in general, are similar to therapeutic gunshots, once the shot hits and interacts with the treated bacterial cell membranes, it forms “pits” that damage the membranes, increasing the permeability of bacterial membranes and killing the bacteria [152]. Then, the Ag NP shots may inactivate the bacterial respiratory chain dehydrogenases, inhibiting respiration [153], and form ROS, damaging the membranes [154,155] (more specific roles of ROS are explained below in Section 5.2. PDT). Factors that affect the bacterial killing efficiency of Ag NP shots remain unclear. The size of the Ag NPs is a fundamental factor. Smaller Ag shots (1 to 10 nm) kill bacteria more efficiently than larger shots [156,157,158]. These small nanoshots have large surface areas that allow larger contact areas with the bacterial cells, killing them [157,159,160].

The immobilization of Ag NP shots on substrates (silica) allows more contact-mode interactions between Ag shots and cells because it inhibits the sequestration of shots within the bacterial cells [161]. Smaller Ag NP shots with high surface areas have higher oxide contents [162]. The oxidized Ag NPs release more Ag^+^ ions [163]. Ag^+^ ions (as such or released from Ag NPs) are soft acids motivated to interact with soft bases containing sulfur and phosphorous. The interaction of Ag^+^ ions with sulfur-containing proteins in bacterial cell walls and thiol groups of bacterial enzymes and proteins inactivates them, damaging bacterial membranes. The interaction of Ag^+^ ions with phosphorus parts of DNA prevents bacterial replication [164,165]. Ag^+^ ions also inhibit the bacterial electron transport chain (which is membrane-bound) via facilitating the oxidation of respiratory membrane-bound enzymes (flavoprotein and cytochrome b1), eventually collapsing bacterial respiration [166,167]. From the mechanisms above, we notice substantial differences and cannot deny the similarities between the antibacterial actions of Ag NPs and Ag^+^ ions. Can we attribute the antibacterial mechanisms of Ag NP shots to Ag^+^ ions? Our previous study has pointed toward the extensive different mechanistic antibacterial actions of Ag NPs and Ag^+^ ions and concluded that Ag NP shots mainly damage the bacterial membranes, whereas Ag^+^ ions mainly target bacterial DNA [158]. A more recent review was dedicated to referring to these substantial differences and similarities between the antibacterial mechanisms of Ag NP shots and Ag^+^ ions. The review extended to shape the formula that not only are the antibacterial mechanisms different, but the bacterial resistance mechanisms against Ag NP shots and Ag^+^ ions would also be different [162].

Would there be antibacterial properties related to the shape of NP shots? Van Dong et al. [168] have highlighted the role of geometric structures (sharp edges and vertices) of triangular silver nanoprisms (broad size range of 25 to 400 nm) in the more prominent antibacterial effects compared with the spherical NPs (even with smaller average sizes of 21 nm). The sharp vertices of Ag nanoprisms disrupt the bacterial membranes, easing the penetration of more Ag prisms into the bacterial cells. In a leading article devoted to stress on the shape-dependent antibacterial effects of Ag NPs [160], truncated triangular silver nanoplates with {111} lattice planes showed the best antibacterial effects against *E. coli* among other shapes of Ag NPs (spheres and rods) and even Ag^+^ ions. The authors also mentioned the sharing of the {111} lattice planes of the truncated triangular nanocrystals in their best antibacterial properties because of the high-atom-density {111} facets, enabling more reactivity with the bacterial cells. Similar effects of sharp edges have been echoed as damaging of bacterial membranes and killing of *S. aureus* and *E. coli* upon direct contact with the extremely sharp edges of the graphene oxide nanowalls. The nanowalls killed more *S. aureus* than *E. coli*, possessing other outer membrane protection against the damage [169].

Finally, what antibiofilm mechanisms might Ag NP shots or NP shots employ in general? Unfortunately, researchers have not studied the interactive mechanisms of NPs with biofilms in much detail. There has also been a controversy between scholars regarding experimental results. Some observers attributed the antibiofilm effects of NPs to the same broad antibacterial mechanisms of NPs (i.e., oxidative stress via ROS, the release of metal ions, and non-oxidative stress). However, this broad concept has been debated by other studies arguing more intricate interactive mechanisms between NPs and biofilms, taking into consideration the formation and architecture of biofilms [170]. Gao et al. [171] identified the retention of catalytic iron oxide NPs within the biofilm architecture of *Streptococcus mutans*. Catalytic NPs activated the co-administered hydrogen peroxide (H_2_O_2_) rapidly under acidic conditions (pH 4.5 to 5.5), mimicking the pH within biofilms and producing in situ free radicals that concurrently degraded the EPS of biofilms and killed the bacterial cells embedded within their biofilms. Ghaseminezhad et al. [172] investigated the antibiofilm effects of Ag NPs and Ag-Fe_3_O_4_ nanocomposites against *S. aureus* biofilms in chronic wounds. The most striking result of their investigation is that the nanocomposites were uniformly distributed within the collagen gel matrix containing deeply embedded biofilms, and *S. aureus* biofilms were eradicated following the administration of the magnetic field, pushing the nanocomposites within the collagen matrix. Meanwhile, Ag NPs could not eradicate *S. aureus* biofilms, despite releasing more Ag^+^ ions and producing more ROS than the nanocomposites. They finally suggested that the antibiofilm mechanisms of their nanocomposites remain murky. Qin et al. [173] have shed light on the molecular antibiofilm mechanisms of Ag NPs immobilized in situ on titanium. They attributed the prolonged inhibition of *Staphylococcus epidermidis* (60 days with seven exposure cycles) to the synergetic (Ag NPs and titanium substrate) inhibition of bacterial adhesion via downregulation of the expression of biofilm-associated genes (*icaA* and *icaD*). These *ica* genes are virulence markers and mediate the full slime (EPS) production of *S. aureus and S. epidermidis* [174]. NPs could inhibit biofilm formation by preventing initial bacterial adhesion by targeting the cell-surface-associated adhesins [170]. The bacterial surface charge is anionic in both Gram-positive bacteria, owing to the contained teichoic acids, and Gram-negative bacteria, owing to the contained lipopolysaccharides in the extra outer membrane [175]. Therefore, NPs could prevent bacterial–surface adhesion and cell–cell adhesion by electrostatic interactions [170]. For instance, carboxymethyl chitosan/amorphous calcium phosphate nanocomplexes decreased the adhesion and biofilm formation of *S. mutans* and *Streptococcus gordonii* on the enamel surface via electrostatic interactions. The cationic amino groups in the nanocomplexes neutralized the anionic bacterial surface charge, decreasing bacterial adhesion to surfaces. The nanocomplexes enhanced the flocculation of particles in the solution, reducing the cell–cell adhesion [176]. Liao et al. [177] have manufactured theranostic Au@Ag core–shell NP-decorated silicon nanowires with therapeutic antibacterial properties against *S. aureus* and *E. coli* via physical stress and chemical effects. Bacterial sensing properties were facilitated in combination with laser-induced breakdown spectroscopy, quantifying the captured bacteria with a low detection limit. Nanowires offered shear stress and binding sites for bacterial cells and large-sized extracellular organelles (e.g., flagella). Au@Ag NPs offered other binding sites for small-sized extracellular organelles (e.g., fimbriae) or membrane proteins. Au cores improved the antibacterial activity of Ag shell atoms. In the same track of preventing bacterial adhesion and biofilm formation, an adhesive containing Ag NP shots releasing Ag^+^ ions inhibited the growth of *S. mutans* biofilms. Ag^+^ ions downregulated *S. mutans* glucosyltransferases (synthesize extracellular glucans essential for bacterial cell adhesion and biofilm formation) gene expressions (*gtfB*, *gtfC*, and *gtfD*) [178]. Ag NP shots could also prevent bacterial biofilm formation by the downregulation of QS-regulated genes, especially those encoding the secretion of virulence factors as detected in *P. aeruginosa* biofilms [179]. We believe that further studies that elaborate on the downregulation by genetic and epigenetic mechanisms in biofilms by NP shots or ions will need to be undertaken.

#### 4.1.3. Theranostic Electrospun Nanofibers

Electrospinning is an economical, practical, and simple method for the preparation of nanofibers [180,181,182,183]. The biological irony is that all human tissues and organs undergo deposition in nanofibrous forms (i.e., bone, dentin, collagen, cartilage, and skin) [184]. Electrospun nanofibers have intriguing properties stemming from (i) their nano-scaled cross-sectional dimensions, such as high surface areas consolidating their functionalization in a straightforward manner (e.g., drugs, ion-exchangers, nanostructures, and NPs), permeability, and porosity [185,186,187,188,189,190]. (ii) Their macroscopic length facilitates a well-appreciated ease of manipulation [189]. These properties of electrospun nanofibers provide a valid account in biosensing [191,192]. Biosensors could provide new vistas in wound care via detecting bacterial wound infections [193]. Electrospun fibers could be a winning strategy in wound dressing applications, hitting both goals of being physical barriers that prevent wound-associated infections and acting as a 3D scaffold that replaces the extracellular matrix (ECM) of the skin of the patient [181,183,185,190]. Alteration in the well-orchestrated skin microbiota composition could be associated with the pathogenesis of inflammatory skin disease [194]. This is seen in chronic colonized wounds with a prolonged inflammatory phase, where bacteria produce inflammatory molecules, attracting inflammatory cells (i.e., neutrophils and macrophages) into the wound. Activated inflammatory cells secrete inflammatory cytokines, inducing the increased production of matrix metalloproteinases (MMPs). The high levels of MMPs degrade the ECM (a gel-like matrix secreted by the cells that it encloses and includes polysaccharides, water, and collagen proteins that elicit the unique elasticity, tensile strength, and compressibility of skin) and consequently impairs healing [139,195]. *S. aureus* is a scourge, commonly colonizing or secondarily infecting the skin of patients with atopic dermatitis [196] and producing delta-toxins that induce allergic immune and inflammatory skin disease [197]. Dermatologists are more inclined to prescribe topical corticosteroids and antibiotics for controlling atopic dermatitis. Phototherapy using ultraviolet (UV; UVA and UVB) is an effective way for controlling atopic dermatitis, especially with unresponsive patients to topical treatments [196]. In a study set out to determine the theranostic approach of fibers, Jin et al. [198] used coaxial electrospinning for preparing core–shell fibers for concurrent imaging and drug delivery. The shell was formed from Eudragit S100 (a pH-sensitive polymer), and the core was formed from poly(ethylene oxide) (PEO) loaded with both the magnetic resonance contrast agent (Gd(III) diethylenetriaminepentaacetate hydrate, Gd(DTPA)) and indomethacin (a non-steroidal anti-inflammatory drug). The cargoes (Gd(DTPA) and indomethacin) were not unloaded in the stomach because of the insolubility of the Eudragit shell in acidic pHs. In contrast, pH increases to alkaline in the intestine’s terminal parts, dissolving the shell. The exposed PEO core to intestinal fluids would swell and adhere to the intestinal walls, unloading the Gd(DTPA) and indomethacin for concurrent imaging and treatment of inflammatory bowel disease, respectively. The pH-responsive drug-loaded electrospun nanofibers could further contribute to controlling implant- and wound-associated infections under the umbrella of nanotheranostics because pH differs in the physiological and pathological milieus of the human body. For example, the acid–base homeostasis maintains the physiological pH of arterial blood in the range of 7.36 and 7.44 [199]. Physiologically, the skin has acidic pH, preventing bacterial colonization [200], whereas chronic wounds and highly infected wounds have alkaline pH (above 7.3). The pH could draw a roadmap, elucidating different phases of the wound healing process that necessitate different ranges of pH [200,201]. Acidic pH in inflamed tissue potentiates the pathogenic endocytosis and phagocytosis by transdifferentiated neutrophils [202]. In another endeavor examining the triggering effect of pH on the drug release from smart electrospun fibers, Yuan et al. [203] have electrospun an ibuprofen-loaded poly(L-lactide) fibrous scaffold and shown a quick ibuprofen release from the scaffold at pH 5.0 (preventing excessive inflammation and promoting muscle wound healing) and a slow-release at pH 7. In an investigation into the antibiotic cargo of electrospun fibers, Alhusein et al. [204] have demonstrated an initial burst release of tetracycline (55%) within the first 3 h followed by a prolonged sustained release (≈80%) after 14 days from a triple (micro/nanofiber)-layered electrospun matrix. The tetracycline-encapsulated electrospun matrix was composed of a central layer of poly(ethylene-co-vinyl acetate) sandwiched within the exterior layers of poly-ε-caprolactone. It eradicated the preformed biofilms of *S. aureus* for three consecutive days. Ag-containing polymeric or composite electrospun fibers could be a major area of interest within fields requiring the prolonged antibacterial actions of Ag NPs, including implant scaffolds [205,206] and wound dressings [207]. The most sought-after properties of ideal wound dressings are (i) facilitating the rapid establishment of homeostasis (the main process toward wound healing); (ii) providing antibacterial properties (preferably in a controlled manner); (iii) preventing wound infections; and (iv) being biocompatible, promoting cell growth. Electrospun nanofibers could elegantly excel wound dressings by incorporating homeostatic agents (e.g., growth factors) and antibacterial agents. Electrospun nanofibers also support homeostasis because of their high surface areas, promoting cell attachment, and their porosities, allowing gaseous exchange and nutrient supply and regulating fluid loss [181,182,190,192,208]. Ag is a forerunner antibacterial agent in electrospun wound dressings because it decreases inflammation at the wound site and enhances epithelialization [188,209], healing, and the cosmetic appearance of skin [210]. Biodegradable polymeric nanofibers could also be directly electrospun on chronic wounds, forming dressings that heal wounds and halt the formation of scar tissue [184,209], as shown in Figure 1. 

### 4.2. Theranostic Antibacterial Star-Shaped Polymers 

The synthesis of star-shaped polymers is an increasingly important area in diagnosis and polymer therapeutics (e.g., drug delivery, antibacterial, and anti-biofilm agents) because of their featured structural (physical and chemical) properties, encapsulation capabilities (because of their 3D structure), functionalities (internal and peripheral), and heightened stimuli-responsiveness [211]. Star polymers can be classified into two types: (i) regular or symmetric star polymers that possess identical arm segments; and (ii) miktoarm star polymers, which are also defined as asymmetric star polymers or heteroarm star polymers, possessing diverse chemical structures, topologies, molecular weights, and functional groups. On the one hand, such heterogeneity of miktoarm star polymers provides the potential to synthesize novel morphological nanostructures and self-assemblies in aqueous media, which is promising for biomedical applications (i.e., drug delivery). On the other hand, the main challenge facing Miktoarm star polymer synthesis is the complicated process to prepare architectures with various precisely designed arms [211,212]. As far as antibacterial properties are intended, the much denser functionalities of star polymers excel in the antibacterial functionalized star polymers over linear polymers’ antibacterial effects [211]. 

The combinations of properties of star polymers provide means for their efficient use for diagnosis in different forms, including fluorescent probes, contrast agents, and in vitro diagnostics [211]. Qiu et al. [213] have shown the thermo-responsive phase transitions of star conjugated copolymers (with different poly(2-(dimethylamino)ethyl methacrylate) (PDMAEMA) chain lengths prepared from the hyperbranched conjugated polymer (HCP)) with adjustable lower critical solution temperature (LCST) according to the pH of the copolymeric solution. By exceeding the LCST, the arms of PDMAEMA were collapsed, and the emission of HCP-star-PDMAEMAs was enhanced, detecting *E. coli* with high sensitivity. Shen et al. [214] fabricated water-soluble fluorescent Ag nanoclusters (i.e., bioimaging labels) from multiarm star poly(acrylic acid) (PAA), which are characterized by strong binding of the protonated acrylic acid groups with Ag^+^. The fluorescence of Ag nanoclusters diminished after 100 min of illumination (i.e., the formation of large Ag NPs). 

**Figure 5:**
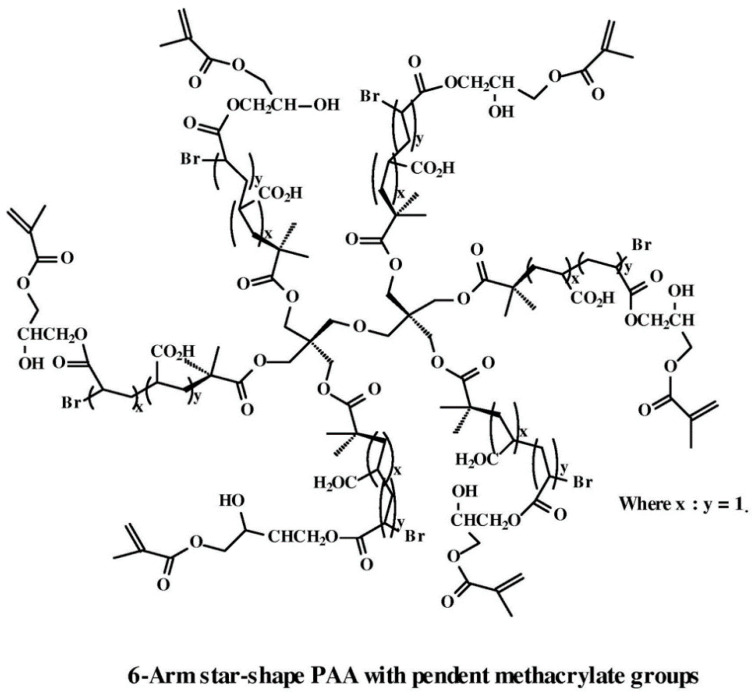
Schematic illustration showing the structure of the six-armed star-shaped poly(AA) with pendent methacrylate groups. Reprinted with permission from [219]. Copyright, 2012, Elsevier.

Sulistio et al. [215] have explored the therapeutic area of star polymers via preparing (in a one-pot approach using amino acid building blocks, N-carboxyanhydrides), a highly functionalized water-soluble and degradable core cross-linked star (CCS) polymers. The therapeutic CCS could release its drug cargo upon degradation. Investigators have recently examined the multimodal antibacterial therapeutic effects of star-shaped peptide polymeric nanoparticles, namely structurally nanoengineered antimicrobial peptide polymers (SNAPPs). SNAPPS combatted multidrug-resistant superbugs via the physical disruption of bacterial cell membranes, dysregulation of ion efflux/influx, and induction of apoptotic-like death [216]. Siedenbiedel et al. [217] identified the antibacterial effects of star-shaped polymers against *S. aureus* and *E. coli*, retaining their antibacterial properties even after 20 water flush treatments. More therapeutic applications of star polymers have also been reported [218], using star-shaped-brush polymers in electrospinning. The electrospun microfibers (100 mg) killed *E. coli* (99%) within 2 h of contact. Weng et al. [219] have shown that furanone containing the star-shaped PAA (as shown in Figure 5) decreased the viability of *Streptococcus mutans*, which was unchanged after 30 days of aging in water, suggesting its potential application as long-lasting antibacterial cement. Bone repair tissue engineering represents a specific application of biodegradable star polymers due to the ease of tuning the mechanical properties and degradation time of star polymers. For example, the covalent cross-links (i.e., the furanone-containing cements with the covalently bonded antibacterial groups mentioned above [219] and in Figure 5) represent a convenient approach to developing star polymers retaining an enhanced long-term application [211] that could reach 30 days in after the furanone-containing cements showed.

### 4.3. Photodynamic Therapy (PDT)

**Figure 6:**
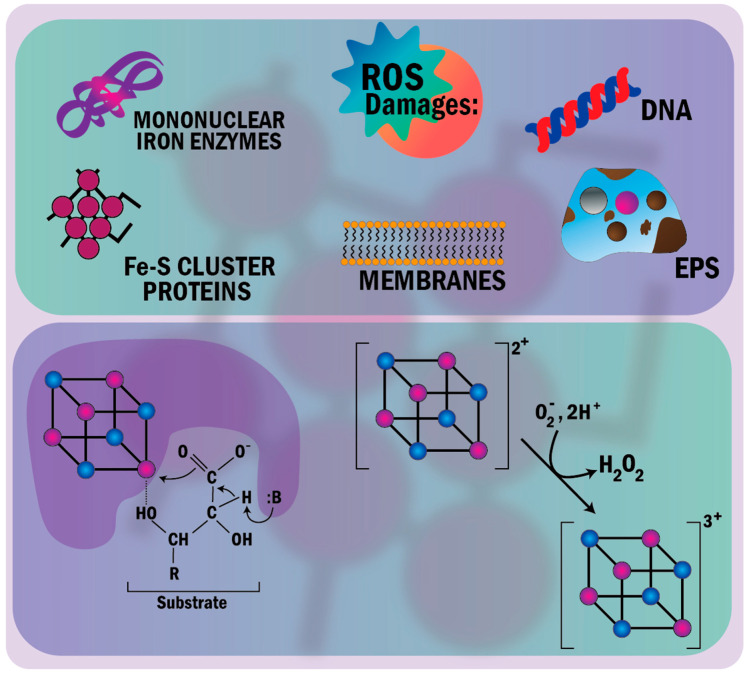
Toxicity mechanisms of reactive oxygen species (ROS). ROS exert multiple roles, damaging (1) bacterial membranes, (2) Fe-S cluster proteins, (3) mononuclear iron enzymes, (4) DNA, and (5) biofilm EPS. A highlighting spot on the damage of iron-sulfur (Fe-S) cluster proteins bound to the α,β-dihydroxy acid substrate after exposure to superoxide (O_2_^−^). The cluster is oxidized, forming hydrogen peroxide (H_2_ O_2_), and converted to the unstable oxidized form [4Fe–4S]^3+^ species [221].

Photodynamic therapy (PDT) plays an essential role in selective bacterial illumination via selective PS and the efficient treatment of bacterial diseases via the produced ROS [108]. The meaning of ROS refers to reduced oxygen metabolites with strong oxidizing abilities, deteriorating cells (i.e., oxidizing proteins and lipids and damaging DNA) at high concentrations, and serving as signaling molecules (i.e., regulating cell growth, differentiation, and apoptosis). Well-known examples of ROS are the superoxide anion (O_2_^•−^), hydroxyl radical (OH^•^), H_2_O_2_, and hypochlorous acid (HOCl) [220]. Three main ROS mechanisms may prove toxic to bacteria (delineated in Figure 6). First, the damage of iron-sulfur (Fe-S) cluster proteins, where O_2_^−^ is electrostatically motivated (because of their chemical nature as univalent electron donors), binds the bacterial solvent-exposed Fe-S cluster. ROS converts them to an unstable oxidized form [221,222] (as displayed in Figure 6). The Fe-S cluster is inherently recruited as an enzymatic cofactor in several cellular processes, such as electron transport and enzymatic catalysis and regulation. The cluster binds α,β-dihydroxy acid substrates, and it is involved in dehydration reactions through catalyzing (activating) dehydratases [223,224]. The decomposition of the unstable oxidized Fe-S cluster inactivates the dehydratases [221]. Bacterial switching to fermentation (anaerobic growth) reduces the damage of the Fe-S cluster [222,223]. The second ROS mechanism involves the damage of mononuclear iron enzymes, where O_2_^−^ and H_2_O_2_ inactivate the enzyme family (i.e., epimerases, dehydrogenases, deformylases, and deaminases) using the single iron atom as an enzymatic cofactor. Ultimately, a third mechanism might be associated with the indirect damage of DNA, where H_2_O_2_ produces OH^•^, damaging the nitrogen-containing nucleobases and deoxyribose of DNA oxidatively [193].

**Figure 7:**
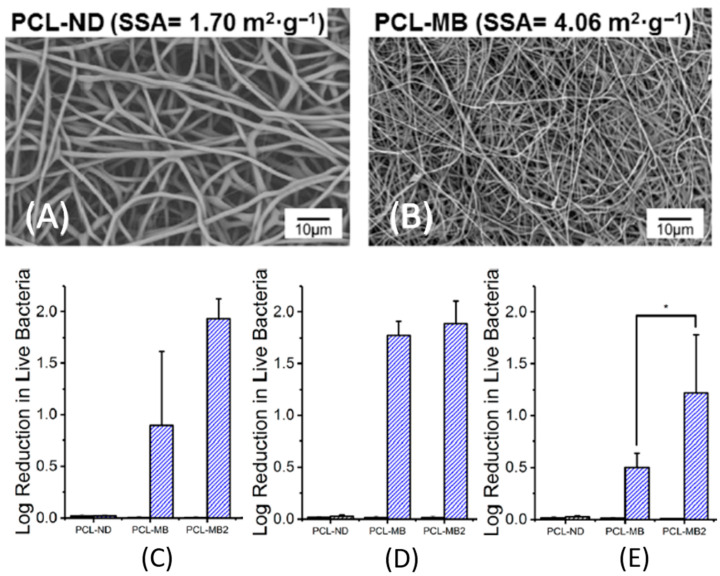
Combined antibacterial electrospun nanofibers and photodynamic therapy (PDT) systems. Scanning electron microscope images of electrospun poly(ε-caprolactone) (PCL) scaffolds without a photosensitizer (PS), (ND) (**A**) and electrospun PCL scaffolds loading a PS (methylene blue, MB) (**B**) at the magnifications of 1000× and with their specific surface areas (SSAs) determined by Brunauer, Emmett, and Teller analysis. Average log reduction of *Escherichia coli* on PS-loaded PCL scaffolds after 30 (**C**), 60 (**D**), and 120 min (**E**) of light exposure. Scaffold denoted PCL-MB2 was electrospun from PCL loaded with a doubled concentration of MB (PS). Black bars denote results obtained after incubation in the dark. Striped bars denote light measurements. Results are represented as means ± SD. The asterisk (*) denotes a significant difference compared with the control (PCL-ND) (*p* < 0.05, Student’s *t*-test). Reprinted with permission from [228]. Copyright, 2019, American Chemical Society.

Zheng et al. [225] have taken advantage of the molecular antibiotic mechanism of MRSA (expressing β-lactamase) and prepared a PS construct (β-lactamase enzyme-activated PS, β-LEAP) that was cleaved by β-lactamase of MRSA. Following the PS cleavage, local ROS were explicitly produced to MRSA upon activation by laser light at 670 nm, inhibiting the growth of MRSA strains. A direct relationship was observed between the β-lactamase activity of MRSA and the susceptibility of MRSA to β-LEAP inducing PDT. Dai et al. [226] have explored the PDT on a bioluminescent MRSA infected mouse wound using a polyethylenimine-ce6 (PS) and a red light. They have observed that PDT inactivated MRSA efficiently, abrogating the bioluminescence of wounds, and enhanced wound healing. However, less PDT inactivation of MRSA would be reached in vivo than in vitro because in vivo, MRSA tends to deeply penetrate tissue, forming biofilms that act as a barrier, hindering PS’s access, and weakening the penetrated light dose into the deep tissue. Jijie et al. [227] have conjugated amine-terminated carbon dots (CDs-NH_2_, size 6 nm) with the carboxy group of ampicillin (AMP) via a crosslinker forming amine-functionalized CDs (CDs-AMP, 41 nm) as a carrier for the immobilization and delivery of AMP cargo. CDs-AMP demonstrated better antibacterial activities than free AMP against *E. coli*. This enhanced antibacterial activity was demonstrated by a decrease (by 40%) in the MIC of immobilized AMP due to the greater exposure of *E. coli* cells to larger molecules of the immobilized AMP that retained its activities even after two weeks of storage (at 4 °C in aqueous solution). CDs-AMP generated ROS (O_2_^•−^) under visible light illumination. The generated O_2_^•−^ increased with greater exposure time and visible light lamp intensity and enhanced the antibacterial activity of the conjugate, disrupting the integrity of *E. coli* membranes. Inspired by the exciting properties of electrospun nanofibers and the antibacterial efficiencies of PDT (without administering antibiotics), Contreras et al. [228] have recently combined both systems. They have encapsulated methylene blue (PS) within a biodegradable electrospun poly(ε-caprolactone) (PCL) nanofibrous scaffold for controlling implant infections. The fibrous scaffold was activated (via visible light) on-demand, reducing the viability of *E. coli* (as a model organism for this combined system) that increased with the time of light exposure (as depicted in Figure 7) due to the production of more ROS. It would be a fruitful area for further work to investigate the combined antibacterial effects of these two systems and different nanotheranostic systems, specifically on MRSA. In the same vein, the fascinating theranostic properties of NPs and nanofibers mentioned above could be combined with PDT, creating a novel theranostic system, including PDT.

### 4.4. Photothermal Therapy (PTT)

PTT is a key technology for the treatment of bacterial diseases. However, PTT remains in a nascent stage (experimentally and only in vitro studies) compared with PDT [108]. Within the PTT technologies, GNPs receive much interest given their elicited optical properties from their plasmon resonance absorption. Different GNPs (spheres, rods, tubes) contribute to the shifting of the absorption band into the near-infrared (NIR) wavelength range and further functionalization [108]. The NIR wavelength range is long (700 to 1700 nm) and is coined as “NIR window” or “optical window” [229,230]. Utilizing this long NIR light-mediated approach has attracted much interest because it allows deep-tissue penetration and minimal photodamage to biological tissue [229,231,232]. In contrast, short-wavelength UV light facilitates both low-tissue penetration and DNA damage, making it unsuitable for clinical therapy [231], and visible light also facilitates low-tissue penetration [229]. GNPs also positively promote the localized thermal damaging effects in the nanometer range surrounding them [108]. 

In PTT studies using GNPs, Zharov et al. [233] developed a theranostic PTT method for the selective laser killing of targeted protein A of *S. aureus* (nanotherapy) by secondary IgG (Immunoglobulin G)-conjugated 40 nm GNPs. A real-time assessment of the nanotherapy was exercised using a photothermal microscope (nanodiagnosis) with higher sensitivity than the transmission technique. Bound *S. aureus* with conjugated GNPS were irradiated with laser pulses (420 to 570 nm, 12 ns, 100 pulses), killing bacterial cells aptly combined with the bubble-formation phenomenon around the clustered GNPs. This apt killing was achieved as increasing the laser energy formed bubbles (life span of 0.1 and 2 ms and size of 1 to 8 μm) around the hot clustered GDNPs, penetrating bacterial cells causing local cell-wall damage and subsequent complete bacterial disintegration. Huang et al. [234] prepared iron oxide (Fe_3_O_4_)@Au nanoeggs and reported a temperature increase in the nanoegg suspension from 23 to about 55 °C after NIR irradiation (808 nm) for 3 min. They have further immobilized vancomycin on the surface of nanoeggs. The treated MRSA were entirely covered with the vancomycin-bound nanoeggs and killed (99%) after NIR irradiation (808 nm, 3 min). The magnetic properties of nanoeggs aggregated the bacterial cells and contributed to an ace photothermal effect of the vancomycin-bound nanoeggs. Wang and Irudayaraj [235] assembled magnetic NPs (MNPs, around 15 nm) surrounding Au nanorods, forming an Fe_3_O_4_-Au_rod_ necklace-like probe. They further functionalized the probe with specific antibodies to *E. coli* and investigated its simultaneous detection, separation, and photokilling properties to *E. coli* within a cocktail of pathogens. The authors recorded a strong longitudinal plasmon for the probe at 765 nm, which decreased after 30 min incubation with *E. coli*. This decrease implied the selective binding of several antibody-bound probes to its targeted much larger *E. coli* cell (around 1 to 3 μm), even at a concentration as low as 10^2^ CFU/mL. Following irradiation with NIR light (785 nm) for 15 min, the probe-bound *E. coli* cells were magnetically separated and cultured, showing no grown colonies and implying probe absorption to adequate energy after excitation in the NIR region killing the targeted *E. coli*.

**Figure 8:**
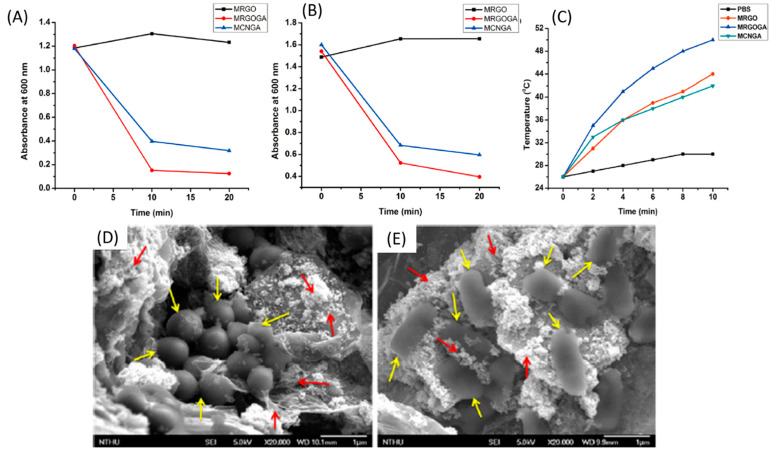
Antibacterial photothermal therapy. Bacterial growth curves measured at optical density 600 nm *Staphylococcus aureus* (**A**) and *E. coli* (**B**). Temperature curves of phosphate-buffered saline (PBS) and 80 ppm magnetic reduced graphene oxide (MRGO), magnetic carbon nanotubes functionalized with glutaraldehyde (MCNGA), and magnetic reduced graphene oxide functionalized with glutaraldehyde (MRGOGA) solutions after near-infrared (NIR) laser (808 nm) irradiation (**C**). SEM images showing MRGOGA (red arrows) captured *S. aureus* (yellow arrows) (**D**) and *E. coli* (yellow arrows) (**E**). Reprinted with permission from [237]. Copyright, 2013, American Chemical Society.

Similar to GNPs, Fe_3_O_4_ MNPs also possess photothermal properties under NIR light illumination, where Tsai-Jung et al. [236] have found that Fe_3_O_4_/alumina core/shell MNPs specifically targeted and inhibited the cell growth of antibiotic-resistant nosocomial bacteria by over 95% within 10 min of illumination. Moreover, the graphene-based PTT was investigated by Wu et al. [237], preparing MNPs (5 to 8 nm) with reduced graphene oxide (sheet-like structure) functionalized with glutaraldehyde (GA, cross-linking or capturing agent), namely MRGOGA. Then, MRGOGA was examined for capturing and killing *S. aureus* and *E. coli* after NIR laser irradiation and in comparison with magnetic reduced graphene oxide (MRGO) and magnetic carbon nanotubes functionalized with GA (MCNGA) (as depicted in Figure 8). They found that PTT was a synergistic interplay of magnetic properties trapping bacterial cells by an external magnet, reduced graphene oxide photothermal properties after irradiation, and GA capturing bacterial cells. They noted no inhibitory effects on bacterial growth for MRGO, showing high bacterial concentrations (measured at an optical density of 600 nm). On the contrary, MRGOGA and MCNGA inhibited bacterial growth, indicating a sharp drop in the bacterial concentrations with more enhanced capturing and killing properties for MRGOGA than MCNGA. After irradiation, MRGOGA rapidly (within 10 min) killed *E. coli* and *S. aureus* via increasing the solution temperature up to ≈50 °C, denaturing bacterial enzymes and proteins and membrane lipids that killed the bacteria. Fan et al. [238] went on to combine the PDT and PTT nanotheranostic systems in a nanoplatform of star-shaped Fe_3_O_4_-Au magnetic core–plasmonic Au shell NPs (≈70 nm) conjugated to methylene blue (PS)-modified aptamer. This combined nanoplatform serves for selective detection and separation, fluorescence imaging, and MRSA destruction within 40 min from binding. Several nanoplatforms are bound to one MRSA cell because of their smaller size (one order of magnitude) than the size of MRSA cells. PTT heat destruction of MRSA cells was facilitated by the induction of plasmonic Au shell by the NIR light (670 nm). The methylene blue developed NIR fluorescence images after magnetic separation (capture efficiency of 96%). Methylene blue simultaneously served as a PS facilitating PDT ROS production after light irradiation and a synergistic agent during PTT, destroying MRSA cells.

## 5. Challenges of Progress 

### 5.1. Bacterial Challenges

The depressing concept of “community prescribing” antibiotics promotes the trend of growing antibiotic resistance and remains futile, given that they are often improperly employed to target viral respiratory infections [239]. The concern of MRSA as a notorious skin and soft-tissue infection could also be extended, even rarely, to community-acquired pneumonia [25]. Culturing methods profiling bacterial communities in chronic wounds are far from ideal detection methods as they overlook an important pathogen such as *Pseudomonas aeruginosa* in chronic wound biofilms [143] and SCVs in patient samples [95]. Moreover, most studies fail to address the relative administration of antibiotics for bacterial persistence and if persisters could trigger the emergence of resistance [84]. The development of effective treatments against biofilms could require adapting targeted treatment strategies against specific bacterial infections instead of a general approach against all biofilms [62,91]. Tailoring a universal surface that prevents the adhesion of different bacterial pathogens, also under different physiological conditions, could be impossible [57]. In contrast, the majority of biofilm infections, especially implant-associated orthopedic biofilms, are a repertoire of multispecies bacterial communities, restraining the efficacy of bacterial species-specific biofilm targeted strategies [4,70].

To date, there is a paradox in the actual antibacterial mechanisms of the action of NPs. Data from several studies suggest that the ROS produced by active NPs (e.g., Ag NPs) are the key players of the antibacterial properties. However, a considerable amount of the literature has refuted any link between the antibacterial properties of NPs and bacterial metabolism. Several other attempts have been made to attribute the antibacterial properties of NPs to a sum of those mentioned above three fundamental mechanisms (oxidative stress, release of metal ions, non-oxidative stress). Therefore, several questions remain to be answered to unravel the antibacterial mechanisms of different NPs in different media conditions using unified standard procedures [47]. If products containing nanotheranostics (e.g., wound dressings) released sub-lethal Ag concentrations, Ag-resistance could be the endpoint [240,241]. Panáček et al. [242] have already demonstrated a bacterial resistance-like mechanism of *E. coli* and *P. aeruginosa* against Ag NPs (28 nm) due to the production of adhesive flagellum protein (flagellin), aggregating Ag NPs to reach 480 nm. Strikingly, the emerged resistance-like mechanism did not involve any genetic change. Ag-resistance genes have also been reported in *Salmonella* plasmid isolated from a hospital burn ward [243] and their homologs in *E. coli* chromosomes [244]. Recently, it was reported that sub-lethal concentrations of copper oxide (CuO) NPs and copper (Cu^2+^) ions mediate the conjugative transfer of plasmid-encoded antibiotic resistance genes from *E. coli* to *Pseudomonas putida*. Horizontal gene transfer was stimulated by ROS (induced by the CuO NPs and ions), damaging bacterial DNA, activating the SOS response, and encouraging the conjugative gene transfer [245]. 

### 5.2. Nanotheranostics’ Challenges

Speaking on the possible induced toxicities by nanotherapeutics, an ideal agent would facilitate selective characteristics for the diseased tissue and a therapeutic effect and demonstrate safety, biodegradability, and lack of immunogenicity [110]. Regarding safety, both the EMA (the regulatory agency assessing medicines for use in the EU for safeguarding human and animal health) and the Food and Drug Administration (FDA) provide similar documentation demonstrating the safety, quality, and efficacy of nanotherapeutics [246]. One of the significant challenges of the theranostic nanomedicine discipline is that most studies are focused on in vitro investigations (e.g., cell culture studies) rather than in vivo investigations to pre-clinical and clinical levels, which are more sophisticated [108,246]. Other potential challenges regarding the targeted delivery of the encapsulated drugs within NPs are (i) achieving successful site-specific drug delivery (lock-and-key concept), (ii) avoiding the premature release of the drug, and (iii) alleviating in vivo cytotoxic effects. These potential challenges could not be mimicked in vitro; thus, the in vitro studies are unsatisfactory [47]. So far, the available in vivo data are limited to either therapeutic or diagnostic investigation instead of their combination. The development of in silico approaches envisaging nanotherapeutics’ biological and toxicological interactions requires comprehensive knowledge on the fate (absorption, distribution, metabolism, and excretion) of the designed nanotherapeutics in vivo and their association with essential commercial features [246]. Possible routes to achieving these challenging approaches will involve (i) forming multidisciplinary research teams; (ii) training to allow nanotherapeutics’ design from different perspectives (engineering, biological, toxicological, and clinical); and (iii) coordination to connect the developed nanotherapeutics in academia with clinical organizations and industrial stakeholders. Another critical aspect that should be resolved is the lack of proper funding to financially support the proof-of-concept of the novel nanotherapeutics designed in academia [47,246]. Furthermore, significant problems of in vivo investigations of nanotheranostics lie in choosing the animal. A classic example of this problem is that rats could self-cure infections without antibacterial help, whereas rabbits could suffer premature death if the challenging bacterial loads were imbalanced. Another well-known example of this problem is that most bacterial infection studies use young, healthy mice or dogs. In contrast, human infections often occur in old, sick, or immune-deficient patients that might also use multiple medications, making no sense to correlate the in vivo animal findings of antibacterial nanotheranostics to the presaged human findings at clinical trials [74]. 

Although electrospun polymeric nanofibers are considered empowered antibacterial wound dressings, incorporating antibacterial agents (via blending followed by electrospinning or core–shell electrospinning or nozzle-free electrospinning) into the nanofibers and their commercialization remain incompetent [182,190]. First, the traditional blending followed by electrospinning stands as unsatisfactory incorporation of the antibacterial agents that remain on the polymeric outer shell and exhibit an unfavorable burst release that could be cytotoxic. Second, in the core–shell electrospinning, the antibacterial agent is encapsulated within a polymeric outer shell that controls its release. However, the high shearing forces (mechanical stresses) employed at the interface between the core and shell fluids, at the initiation of electrospinning, could deteriorate the bioactive agents [182,190,247]. Third, the nozzle-free system simplifies the electrospinning (without maintaining any Taylor cones). Nevertheless, this system requires intricate maintenance of the high voltage that could eventually generate sparks [182]. Fourth, a lack of congruence to reproduce identical scaffolds, especially between diverse research groups, limits the tissue-engineering applications of the electrospun fibrous mats. [182]. Fifth, even with the ongoing research for the antibacterial electrospun nanofibers, data about their in vivo safety assessments are limited [182,183,192]. Therefore, even with the agile features of biopolymeric electrospun nanofibers that could shine in wound dressing applications, the electrospun fibers’ biocompatibility remains a potential issue. This issue is due to the possible presence of impurities (e.g., residual solvents and linkers) that can elicit immunological responses [182,190].

The biostability and biodegradability of star polymers are essential criteria that should coincide with their potential applications. The perfect star polymers are stable in maintaining their functions and then sharply biodegrade into non-toxic small molecules that could be eliminated from the body. The tactics for tailoring the biodegradability of star polymers are (i) synthesizing biodegradable arms via using biodegradable polypeptides, polymers (e.g., poly(d,l-lactide) and PCL), and cleavable linkers; (ii) manufacturing biodegradable cores via biodegradable multi-functional molecules (e.g., cyclodextrin and dextrin) and cleavable linkers; and (iii) compiling biodegradable arms, cores, and linkers [211]. A potential problem of PDT technology, using a PS construct based on the molecular target of bacterial resistance, would be the targeted light delivery to the infected site to activate PS to produce local ROS. However, fiber optics could open vistas for the targeted light delivery to specific infected sites [225]. A future greater focus on investigating the role of NPs as imaging contrast agents or specifically combining contrast-enhanced diagnostic imaging with PDT could produce interesting findings.

## 6. Our Perspectives: What Solutions?

As hospital settings’ role as a source of nosocomial HA-MRSA infections has been outlined above, combating MRSA infections could be achieved by limiting unnecessary hospital visits and performing hand hygiene that is shown to be effective in preventing MRSA. Applying measures, such as isolating MRSA infected patients, using protective gear, reducing inappropriate administration of broad-spectrum antibiotics, and screening MRSA and eradicating MRSA in colonized patients and healthcare workers should be adequate measures to curb MRSA infections [37]. We propose a need for legislation preventing the over-the-counter acquisition of antibiotics, especially in developing countries, to decrease the pace of emergent resistance. In addition, since NPs have multiple simultaneous antibacterial mechanisms, they paralyze the ability of bacteria to mutate and develop resistance against them genetically. Therefore, we also propose taking the nanotheranostics and their combinations with antibiotics into consideration for development, safety assessment, and approval as theranostics against MRSA infections.

Regarding the biofilm dilemma, understanding the mechanism behind how persisters of *S. aureus* form would improve our ability to control chronic tolerant infections [88] instead of removing the untreated infected medical implant or sharp debridement of the wound. In our words, “we must be intelligent, by acquiring knowledge, to best deal with the intelligence of bacteria such as forming tolerant persisters.” For example, considering the dependence of the formation of persisters on (p)ppGpp signaling, using (p)ppGpp synthesis inhibitors, namely relacin, has shown an elegant reduction of the cell viability and disruption of biofilms [248]. The acyldepsipeptide antibiotic (ADEP4) activating ClpP protease has demonstrated efficient killing of persister cells via degrading over 400 proteins, forcing the self-digestion of cells [249]. Adding glucose to treated *S. aureus* persisters with daptomycin has also shown a five-fold increase in the killing of persisters in one hour. This synergistic glucose–daptomycin effect could be attributed to glucose induction to specific carbohydrate transport proteins, increasing the susceptibility to daptomycin, or to glucose stimulating the release or activity of cell-lytic proteins, enhancing the action of daptomycin [250]. Therefore, taking advantage of such agents (relacin, ADEP4, and glucose while considering glycemia) to be used as adjuvants with antibiotics or as cargoes in nanotheranostic systems could provide a brilliant regime for fighting tolerant and resistant chronic infections.

We believe that electrospun wound dressings could serve as multi-functional smart dressings, controlling atopic dermatitis. We propose using star polymers with UV-light responsiveness for electrospinning, where we can benefit from UV-light for both phototherapy and controlled unloading of corticosteroids and antibiotics as an effective topical treatment of atopic dermatitis. If researchers incorporated NPs mediating the diagnosis of *S. aureus* into such electrospun wound dressings controlling atopic dermatitis, then the successful development of what we call “multifunctional theranostic nanofibers” would be achieved. 

We are coming now to the critical point of biocompatibility and safety of nanotheranostics, which could be comprehensively reviewed in a separate manuscript. A recent review has highlighted that every distinct form of Ag NP is considered a separate compound with distinctive physicochemical properties and unique antibacterial and resistance mechanisms [162]. We would like to expand this perspective to infer that every nanotheranostic system is a unique product that should be thoroughly investigated for biocompatibility and approved before any commercial antibacterial theranostic application.

## 7. Conclusions and Future Directions

Here, we have focused on deciphering the problematic *S. aureus* resistance and biofilm (lifestyle and myths surrounding it) clinical infections associated with implants and wounds. Numerous promising antibacterial nanotheranostic systems have been described in this comprehensive review. Whereas the literature is beginning to scratch the surface of the working mechanisms of antibacterial nanotheranostics, this tutorial review has unraveled the detailed antibacterial and antibiofilm mechanisms of nanotheranostics, allowing efficient therapy and rapid diagnosis to curb *S. aureus* infections competently. We have sought to provide one of the first attempts to thoroughly criticize the reviewed literature and provide detailed appraisals for combating *S. aureus* infections. Our “three action appraisals” are are follows. (i) First, there are preventive actions that would have two wings. The first general wing includes avoiding unnecessary hospital visits, hand hygiene, and legislations against over-the-counter antibiotics, especially in developing countries. Our second wing of recommended prevention actions includes preventing the adverse side effects of the NPs from resistance and toxicity. On the one hand, preventing developed resistance against NPs could be started by establishing standard testing procedures and breakpoints of bacteria’s susceptibility to NPs, simulating the standard breakpoints of antibiotic susceptibilities. On the other hand, the toxicity should be avoided by dealing with every single batch of synthesized NPs as a separate material that should be thoroughly examined for toxicity in vitro and in vivo, following standard toxicity tests for NPs. (ii) Theranostic actions include considering NPs combined with antibiotics, glucose, or antiviral drugs, and “multifunctional theranostic nanofibers” to diagnose and treat *S. aureus* infections. We believe in the promising feasible application of these multifunctional theranostic nanofibers in a short time. This belief is based on the fact that the theranostic nanofibers would be applied topically, for example, on wound dressings, against the notorious skin pathogen *S. aureus*. This topical application would decrease the burden of safety issues for NPs without neglecting the necessary toxicity tests that would remain crucial before applications. (iii) Scientific actions include scientific collaborations between multidisciplinary scientists and the international implementation of these appraisals, especially establishing standard testing procedures to close the open uncertainty loop surrounding NPs and their further application as nanotheranostics. 

A fruitful area for further work would be the implementation of PDT and PTT systems in photodynamic therapy and radiotherapy directed for cancer treatment. We also want to direct the reader’s attention to the following recent, authoritative, and interesting literature to considerably investigate other antibacterial and anti-biofilm drug delivery or nanotheranostic systems (e.g., liposomes [251,252], polymeric nanosystems [253,254], and chitosan-based NPs [255,256]) that constitute importance and, unfortunately, remain out-of-the scope of the present work. This work’s natural forward movement is to comprehensively review the potential anti-coronavirus properties of nanotheranostics as a smart solution containing the apocalyptic coronavirus disease 2019 (COVID-19) pandemic and to unravel the MRSA burden on co-infections with COVID-19. This burden needs to be elucidated, considering the association of resistant MRSA producing Panton-Valentine leukocidin toxins with pneumonia. Moreover, we are currently finalizing our extended work on reviewing the safety concerns associated with the administration of antimicrobial nanotheranostics to unveil the answer to the alarming question: Would nanotheranostics be safe? We would further conduct empirical research, based on our critical experience, on biocompatible antibacterial (anti-*S. aureus*) and anti-coronavirus nanotheranostics to determine the clinical efficacy of such systems. Ultimately, we propose the promising employment of nanotheranostics with their detection and detailed antibacterial and antibiofilm properties to curb *S. aureus* and their biofilms in different fields where they are implicated. Such employment could span across the medical, environmental, and agricultural sectors.

## Abbreviations

ADEP4, acyldepsipeptide antibiotic; *agr*, accessory gene regulator; AMP, Ag, silver; ampicillin; β-LEAP, β-lactamase enzyme-activated PS; BR, breast reconstruction; CA-MRSA, community-acquired MRSA; CCS, core cross-linked star; CDs-NH_2_; amine-terminated carbon dots; Cu-MOF, copper-based metal-organic framework; CuO, copper oxide; DNA, deoxyribonucleic acid; (e-DNA), extracellular deoxyribonucleic acid; ECM, extracellular matrix; EMA, European Medicines Agency; EPS, extracellular polymeric substance; Fe-S, iron-sulfur; FDA, Food and Drug Administration; GA, glutaraldehyde; Gd(DTPA), Gd(III) diethylenetriaminepentaacetate hydrate; GNPs, gold nanoparticles; HA-MRSA, hospital-acquired-MRSA; HCP, hyperbranched conjugated polymer; LCST, lower critical solution temperature; MCNGA, magnetic carbon nanotubes functionalized with GA; MNPs, magnetic NPs; MRGO, magnetic reduced graphene oxide; MRGOGA, magnetic reduced graphene oxide functionalized with GA; MRSA, methicillin-resistant *S. aureus*; MSNs, mesoporous silica NPs; NIR, near-infrared; NPs, nanoparticles; PAA, poly(acrylic acid); PBP, penicillin-binding protein; PCL, poly(ε-caprolactone); PDT, photodynamic therapy; PEO, poly(ethylene oxide); PJIs, prosthetic joint-associated infections; PMMA, polymethylmethacrylate; (p)ppGpp, guanosine pentaphosphate; PS, photosensitizer; PTT, photothermal therapy; PDMAEMA, poly(2-(dimethylamino)ethyl methacrylate); QDs, quantum dots; QS, quorum sensing; ROS, reactive oxygen species; *SCCmec*, Staphylococcal cassette chromosome *mec*; SCVs, small-colony variants; SNAPPs, structurally nanoengineered antimicrobial peptide polymers; TA, toxin-antitoxin; 3D, three-dimensional; VRE, vancomycin-resistant enterococci; UV, ultraviolet; VRSA, vancomycin-resistant *S. aureus*.
